# Real-world comparative effectiveness and safety of tofacitinib and baricitinib in patients with rheumatoid arthritis

**DOI:** 10.1186/s13075-021-02582-z

**Published:** 2021-07-23

**Authors:** Naoki Iwamoto, Shuntaro Sato, Shota Kurushima, Toru Michitsuji, Shinya Nishihata, Momoko Okamoto, Yoshika Tsuji, Yushiro Endo, Toshimasa Shimizu, Remi Sumiyoshi, Takahisa Suzuki, Akitomo Okada, Tomohiro Koga, Shin-ya Kawashiri, Keita Fujikawa, Takashi Igawa, Toshiyuki Aramaki, Kunihiro Ichinose, Mami Tamai, Hideki Nakamura, Akinari Mizokami, Tomoki Origuchi, Yukitaka Ueki, Katsumi Eguchi, Atsushi Kawakami

**Affiliations:** 1 Department of Immunology and Rheumatology, Division of Advanced Preventive Medical Sciences, Nagasaki University Graduate School of Biomedical Sciences, Nagasaki, Japan; 2 Clinical Research Center, Nagasaki University Hospital, Nagasaki, Japan; 3 Department of Rheumatology, Sasebo City General Hospital, Sasebo, Japan; 4 Department of Rheumatology, Japanese Red Cross Nagasaki Genbaku Hospital, Nagasaki, Japan; 5 Center for Bioinformatics and Molecular Medicine, Nagasaki University Graduate School of Biomedical Sciences, Nagasaki, Japan; 6 Departments of Community Medicine, Division of Advanced Preventive Medical Sciences, Nagasaki University Graduate School of Biomedical Sciences, Nagasaki, Japan; 7 Department of Rheumatology, Japan Community Healthcare Organization, Isahaya General Hospital, Isahaya, Japan; 8 Department of Rheumatology, Sasebo Chuo Hospital, Sasebo, Japan; 9 Department of Physical Therapy, Nagasaki University Graduate School of Biomedical Sciences, Nagasaki, Japan

**Keywords:** Rheumatoid arthritis, Tofacitinib, Baricitinib, Comparison, Molecular-targeted therapy

## Abstract

**Objective:**

To compare the efficacy and safety of tofacitinib and baricitinib in patients with RA in a real-world setting.

**Methods:**

A total of 242 patients with RA who were treated with tofacitinib (*n* = 161) or baricitinib (*n* = 81) were enrolled. We evaluated efficacy and safety between tofacitinib and baricitinib using multivariable analyses to avoid confounding. Their clinical disease activity and AEs were evaluated for 24 weeks.

**Results:**

The mean (SD) DAS28-ESR change from baseline to 24 weeks was 1.57 (1.55) (tofacitinib) and 1.46 (1.36) (baricitinib). There was no significant difference in the clinical response between the two groups (adjusted mean difference, 0.04; 95% CI, −0.35 to 0.28). The efficacy was not significantly changed in the patients without concomitant MTX use in both groups, but the concomitant MTX use showed better clinical efficacy in the cases of baricitinib treatment. In both groups, the most common AE was herpes zoster infection, and the AE rates were similar between the two groups. However, the predictive factors contributing to clinical response as revealed by a multivariable logistic analysis differed. The concomitant oral steroid use was independently associated with the achievement of DAS-low disease activity in the tofacitinib group, whereas in the baricitinib group, the number of biological and/or targeted synthetic DMARDs previously used was associated.

**Conclusions:**

Our findings indicate that tofacitinib and baricitinib had comparable continuing efficacies and safety profiles. However, there is a possibility that the influence of clinical characteristics on the treatment response differs. The comparison provides useful information to the optimal use of JAK inhibitors in real-world settings.

**Supplementary Information:**

The online version contains supplementary material available at 10.1186/s13075-021-02582-z.

## Introduction

Rheumatoid arthritis (RA) is a chronic autoimmune disease characterized by chronic synovitis that symmetrically develops in joints, and persistent inflammation in joints leads to the destruction of joints and tendons, resulting in deformities and ankylosis. Rheumatoid arthritis affects approx. 0.5–1% of the population worldwide. The use of biological disease-modifying antirheumatic drugs (bDMARDs)—which are able to selectively interfere with a specific molecule such as tumor necrosis factor-alpha (TNF-α) or a cellular pathway such as T-cell activation—enables the achievement of low disease activity or even remission in a high percentage of cases [1, 2]. In addition to bDMARDs, recently, Janus kinase (JAK) inhibitors can be used to treat RA and are beginning to play a crucial role in the management of RA [3]. Because the JAK pathway is involved in many biologic functions (including the activation of the inflammatory cascade in immune cells) and is associated with several cytokines that are closely related to the pathogenesis of RA [4], blockade of the JAK pathway is effective in RA treatment.

Tofacitinib is a non-selective first-generation JAK inhibitor that acts by inhibiting JAK1, JAK2, JAK3, and to a lesser extent TYK2. Six pivotal randomized phase III clinical studies demonstrated RA patients’ good treatment response to tofacitinib among patients with differing statuses such as methotrexate (MTX)-naïve subjects, and the studies revealed the patients’ inadequate response to MTX/bDMARDs [5–10]. Moreover, several studies in real-world settings indicated that tofacitinib was as effective as was observed in these phase III trials [11, 12].

After the approval of tofacitinib in 2012 (USA) and 2013 (Japan), baricitinib was approved for the treatment of RA in 2018 (USA) and 2017 (Japan). Baricitinib prevents the activation of JAK1 and JAK2. As with tofacitinib, the clinical efficacy of baricitinib was assessed by several randomized phase III clinical studies, and these studies showed the usefulness of baricitinib as a mono- or combo-therapy for patients with RA [13–16]. Regarding the two drugs’ pharmacological action, baricitinib acts more selectively on JAK1 than tofacitinib, and for example, baricitinib thus strongly prevents STAT phosphorylation by interleukin (IL)-6 [17]. On the other hand, tofacitinib acts on JAK3, which baricitinib cannot inhibit. Considering such differences, the treatment response might be different even between these two JAK inhibitors. However, until now, no published data of a direct comparison among JAK inhibitors in RA have been available, and the efficacy and safety of these JAK inhibitors, especially baricitinib, in a real-world setting have rarely been described. It is important to determine the differences and similarities of these JAK inhibitors in a real-world setting for the treatment of RA so that the optimal agent can be administered in each case or population.

Direct comparisons of clinical efficacy among treatments are generally scarce in randomized controlled trials (RCTs), especially in RA treatment. However, by appropriately controlling confounding, the data from observational studies can be used for a comparison. We conducted the present study to compare the efficacy and safety of tofacitinib with those of baricitinib by comparing multiple variable-adjusted estimates in a real-world setting. We also analyzed the respective factors that contribute to the clinical response to each of these JAK inhibitors.

## Patients and method

### Patients

All patients were registered in this study at one of the following institutions: the Department of Immunology and Rheumatology, Nagasaki University Graduate School of Biomedical Sciences; Sasebo Chuo Hospital, Isahaya General Hospital, Sasebo City General Hospital, and the Japanese Red Cross Nagasaki Genbaku Hospital. A total of 242 patients who were treated with tofacitinib (*n* = 161) between August 2013 and October 2019 or baricitinib (*n* = 81) between January 2018 and February 2020 were enrolled. All patients had a diagnosis of RA based on the 2010 American College of Rheumatology (ACR)/European League against Rheumatism (EULAR) classification criteria for RA [18].

We collected the enrolled patients’ data at the initiation of each treatment, including the disease duration, positivity of rheumatoid factor (RF) and anti-citrullinated protein antibodies (ACPA), modified Health Assessment Questionnaire (mHAQ), history of previous disease-modifying antirheumatic drugs (DMARDs), and concomitant medications. The treatment with tofacitinib or baricitinib was administered by the patients’ attending rheumatologists in accord with the Japan College of Rheumatology (JCR) guidelines. The patients received either 5 mg of tofacitinib twice/once daily (in patients with renal impairment) daily or 4 mg/2 mg (in patients with renal impairment) of baricitinib once daily with no change in any concomitant csDMARD therapy during the 24-week observation period. The patients gave their informed consent to be subjected to the protocol, which was approved by the Institutional Review Board of Nagasaki University (IRB approval no. 11032819).

### Clinical efficacy and safety

The patients’ clinical disease activity was assessed using the Disease Activity Score in 28 joints-erythrocyte sedimentation rate (DAS28-ESR), Simplified Disease Activity Index (SDAI), and Clinical Disease Activity Index (CDAI) at the baseline and at 4, 8, 12, 16, 20, and 24 weeks after the initiation of tofacitinib or baricitinib treatment. Safety was also assessed based on the adverse events (AEs) reported by the patients as well as on the findings of physical examinations until 24 weeks.

### Statistical analysis

We summarized and compared the baseline demographic and disease characteristics between the baricitinib-treated and tofacitinib-treated patients. The continuous variables were compared by Wilcoxon’s rank sum test and are presented as the medians with interquartile range (IQR). The categorical variables were compared by Fisher’s exact test and are presented as the number of patients and percentages.

For the evaluation of efficacy, first, we conducted a mixed effect model with a repeated measures analysis of variance (ANOVA) to ascertain whether there were significant differences in clinical efficacy between the two treatment groups during the treatment period. The model included the treatment group, treatment period, baseline efficacy, and the multiple term of group and period; confounders included sex, age, disease duration, MTX use, oral steroid use, the number of previous biological or/and targeted synthetic (b/ts) DMARD use, DAS28-ESR, SDAI, mHAQ, presence of RF, and ACPA. Second, the disease activities during week 24 were evaluated risk, crude risk ratio, and adjusted risk ratio, which was estimated by the modified Poisson regression model [19]. The model included the treatment group and above-mentioned confounders. Here, “risk” refers to a remission rate or a rate of patients who achieved less than low disease activity. We used the last observation carried forward (LOCF) method for patients who withdrew before week 24 and in cases of missing data. Third, we summarized the disease activity between groups with and without MTX treatment by percentage and compared the values with Fisher’s exact test.

For the evaluation of safety and drug retention rate, we first summarized and compared the adverse events. We then estimated the drug retention rate in each group by the Kaplan-Meier method and compared them by the Cox proportional hazard model, which is the same as the modified Poisson regression model above.

Finally, we evaluated the predictive factor of clinical responses by performing univariate and multivariable logistic regression analyses. Variables with *P* values < 0.1 in the univariate logistic regression analyses were entered in the multivariable logistic regression analysis. The statistical significance for all tests was defined by a two-tailed *P* value < 0.05. We calculated a 95% confidence interval (95% CI) for each estimate. Analyses were performed using R ver. 4.0.2 (R Foundation for Statistical Computing, Vienna, Austria), GraphPad Prism software (GraphPad Software, San Diego, CA), and JMP Statistical Software (SAS Institute, Cary, NC).

## Results

### Baseline characteristics

The patients’ baseline demographic and disease characteristics are summarized in Table 1. The tofacitinib-treated patients had worse disease activity scores, longer disease durations, more concomitant use of MTX, and lower RF positivity compared to the baricitinib-treated patients. However, the difference was significant only in the rate of a concomitant use of MTX. The patients treated with tofacitinib exhibited high-moderate disease activity (DAS28-ESR 5.17, SDAI 20, CDAI 19) with a median age of 67 years and median disease duration of 12 years. The patients treated with baricitinib also exhibited high-moderate disease activity (DAS28-ESR 5.13, SDAI 19, CDAI 18) with a median age of 66 years and median disease duration of 11 years. The concomitant use of MTX at baseline was present in 68% of the tofacitinib group and 47% of the baricitinib group. The concomitant use of an oral steroid was present in 53% of the tofacitinib group and 47% of the baricitinib group. Approximately 80% of the patients in both groups had been treated with a b/ts DMARD (the median number of previous uses of b/tsDMARDs was 2 in both groups). Regarding switching from another JAK inhibitor, none of the patients in the tofacitinib group had been treated with another JAK inhibitor, whereas 26 of the patients in the baricitinib group had been treated with tofacitinib.

**Table 1:** Clinical characteristics of the study population

	Tofacitinib (*n* = 161)	Baricitinib (*n* = 81)	*P* value
Female, *n* (%)	133 (82.6)	68 (84.0)	0.857
Age (years)	67 [58–73]	66 [56–74]	0.667
Duration of RA (years)	12 [6–18]	11 [4–18]	0.243
Concomitant MTX use, *n* (%)	109 (67.7)	37 (45.7)	0.001
Mean MTX dose (mg/week)	8.62 ± 2.49	8.05 ± 2.69	0.427
Concomitant oral steroid use, *n* (%)	86 (53.4)	38 (46.9)	0.345
Mean oral steroid dose (mg/day)	4.80 ± 2.72	4.80 ± 3.08	0.844
ACPA positive, *n* (%)	124 (77.0)	65 (80.2)	0.624
RF positive, *n* (%)	122 (75.8)	70 (86.4)	0.064
No prior use of b/tsDMARDs, *n* (%)	37 (23.0)	18 (22.2)	> 0.999
Number of previous use of b/tsDMARDs	2.00 [1.00–3.00]	2.00 [1.00–3.00]	0.968
DAS28-ESR	5.17 [4.08–6.11]	5.13 [4.21–5.98]	0.622
SDAI	20 [14–32]	19 [14–29]	0.507
CDAI	19 [12–30]	18 [12–27]	0.497
mHAQ	0.60 [0.1–1.3]	0.62 [0.12–1.38]	0.372

Data are median [interquartile range] unless otherwise indicated

*RA* rheumatoid arthritis, *MTX* methotrexate, *ACPA* anti-citrullinated protein antibodies, *RF* rheumatoid factor, *b/tsDMARDs* biological and/or targeted synthetic disease-modifying antirheumatic drugs, *DAS* disease activity score, *ESR* erythrocyte sedimentation rate, *SDAI* Simplified Disease Activity Index, *CDAI* Clinical Disease Activity Index, *mHAQ* modified Health Assessment Questionnaire

### Clinical efficacy

**Fig. 1:**
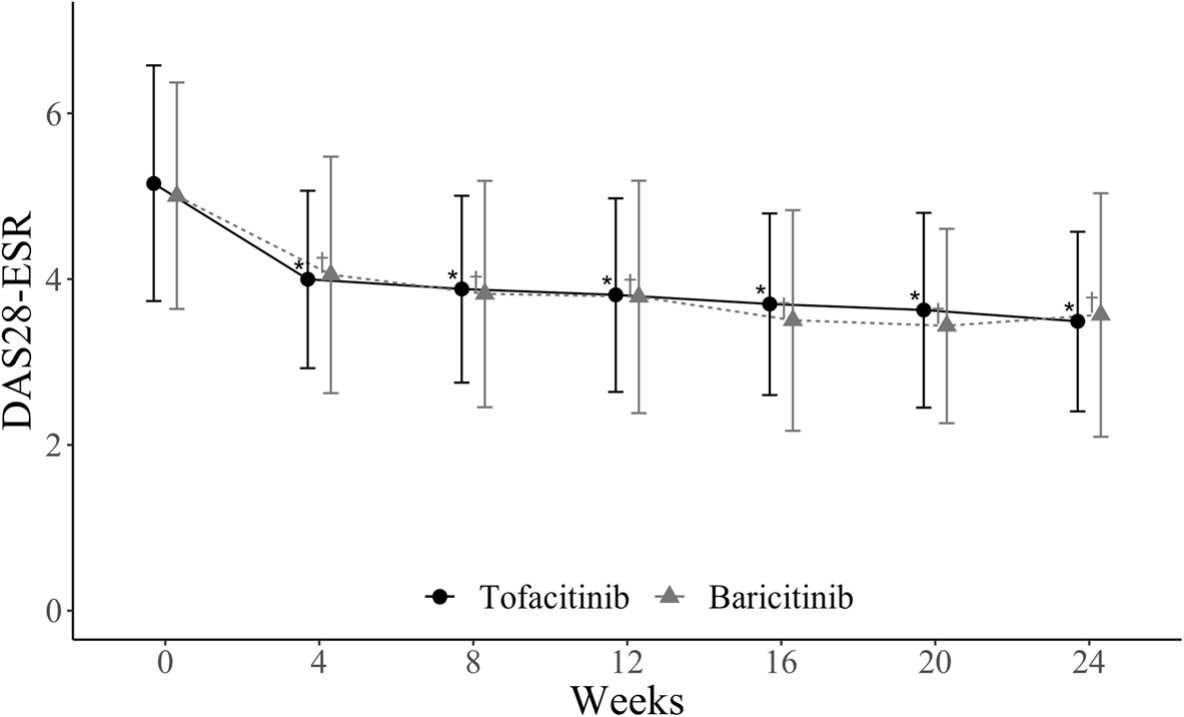
Time course of disease activity scores over 24 weeks of tofacitinib and baricitinib treatments. *Points* and *bars* represent means and standard deviations, respectively. ^*,†^*P* < 0.0001 vs baseline by the Wilcoxon signed rank test. *ESR* erythrocyte sedimentation rate, *DAS* disease activity score

Figure 1 illustrates the changes in the DAS28-ESR values over the 24-week study period. The mean (standard deviation (SD)) DAS28-ESR score for the tofacitinib-treated patients decreased significantly from 5.16 (1.42) at baseline to 3. 29 (1.08) at 24 weeks; the mean (SD) of change from baseline to 24 weeks was 1.57 (1.54). The baricitinib-treated patients also showed a significantly improved DAS28-ESR from 5.01 (1.37) at baseline to 3. 57 (1.47) at 24 weeks; the mean (SD) of change from baseline to 24 weeks was 1.46 (1.36). The adjusted difference mean at 24 weeks between tofacitinib and baricitinib was −0.04 (95% CI, −0.35 to 0.28). At the 24-week follow-up, the DAS28-ESR, SDAI, and CDAI remission rates were 18.0%, 21.1%, and 18.0% in the tofacitinib group and 24.7%, 27.2%, and 22.2% in the baricitinib group, respectively. The rates of patients who achieved less than low disease activity of each clinical indicator (DAS28-ESR, SDAI, CDAI) were 31.7%, 64.0%, and 65.8% in the tofacitinib-treated group and 45.7%, 61.7%, and 60.5% in the baricitinib-treated group (Table 2). Adjusted remission rate also showed the same pattern. There was no significant difference in the clinical responses of the baricitinib-treated patients and the tofacitinib-treated patients. These results suggest that the efficacy of tofacitinib for RA over a 24-week period and that of baricitinib were similar in daily clinical practice.

**Table 2:** Disease activity at 24 weeks

	Tofacitinib (*n* = 161)	Baricitinib (*n* = 81)	Crude risk ratio (95% CI)	Adjusted risk ratio (95% CI)
**DAS28-ESR**				
Remission, *n* (%)	29 (18.0)	20 (24.7)	0.73 (0.44 to 1.21)	0.71 (0.44 to 1.18)
LDA achievement, *n* (%)	51 (31.7)	37 (45.7)	0.69 (0.50 to 0.96)	0.68 (0.49 to 0.95)
**SDAI**				
Remission, *n* (%)	34 (21.1)	22 (27.2)	0.78 (0.49 to 1.24)	0.73 (0.46 to 1.16)
LDA achievement, *n* (%)	103 (64.0)	50 (61.7)	1.04 (0.84 to 1.27)	1.03 (0.85 to 1.24)
**CDAI**				
Remission, *n* (%)	29 (18.0)	18 (22.2)	0.81 (0.48 to 1.37)	0.80 (0.47 to 1.37)
LDA achievement, *n* (%)	106 (65.8)	49 (60.5)	1.09 (0.88 to 1.34)	1.11 (0.91 to 1.35)

Adjusted risk ratios were estimated using a modified Poisson regression model, which included the treatment group (tofacitinib vs baricitinib) and confounders as sex, age, disease duration, MTX use, oral steroid use, the number of previous biological/target synthetic DMARD use, DAS28-ESR, SDAI, mHAQ, presence of RF, and ACPA

*DAS* disease activity score, *ESR* erythrocyte sedimentation rate, *LDA* low disease activity, *SDAI* Simplified Disease Activity Index, *CDAI* Clinical Disease Activity Index, *CI* confidence interval

We next analyzed the efficacy of each of the JAK inhibitors in subgroups divided by the patients’ concomitant use/non-use of MTX. Although age and mHAQ in both groups, disease duration, and history of b/ts DMARD use in the tofacitinib group were different, the baseline variables including disease activity of the subgroups were almost equal regardless of MTX use/non-use (Suppl. Table S1). In the tofacitinib group, the mean DAS28-ESR change from baseline to 24 weeks was 1.42 in the patients with concomitant use of MTX and 1.07 in those without concomitant use of MTX, whereas in the baricitinib group, the corresponding values were 1.47 and 0.89, respectively.

**Fig. 2:**
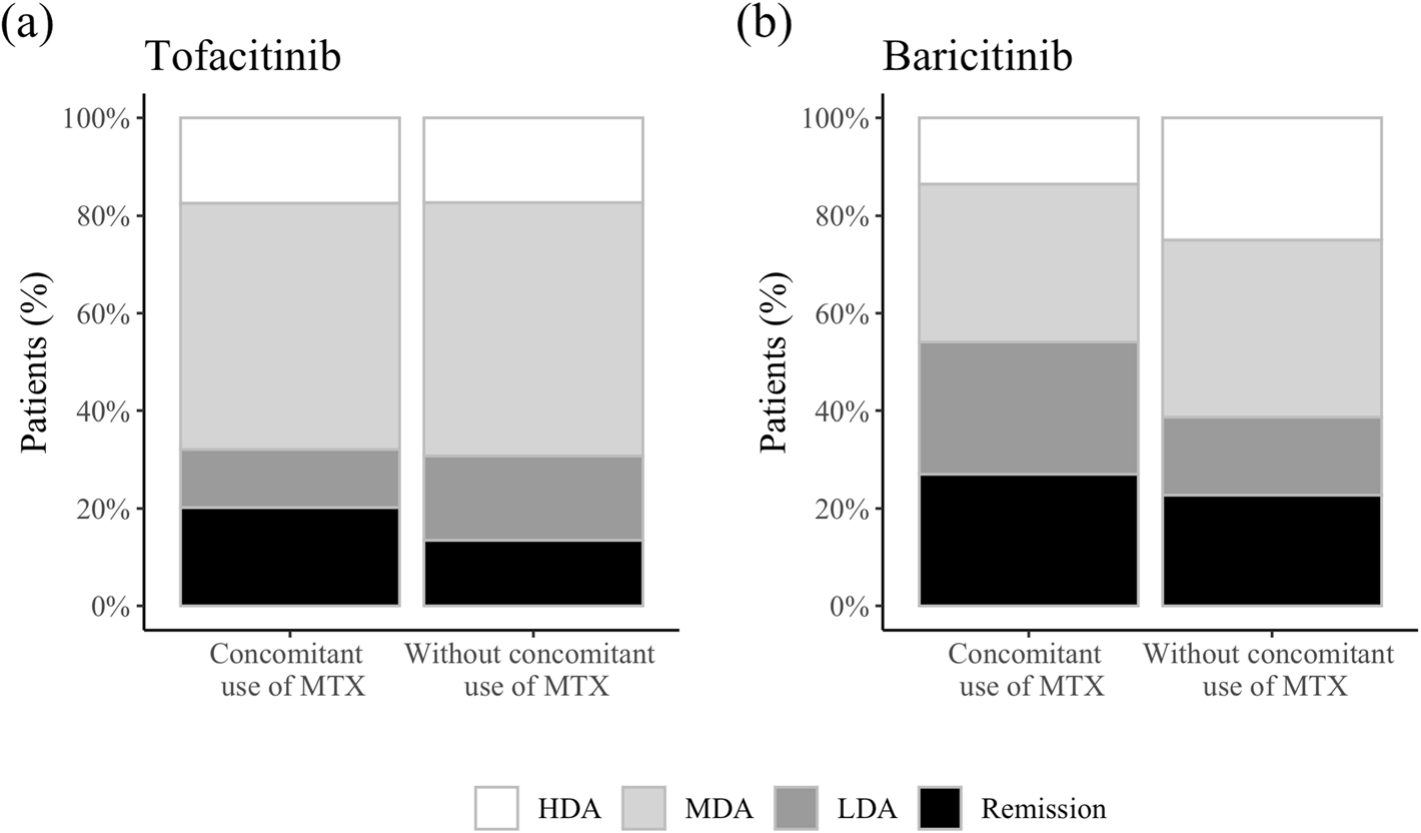
**a** The proportion of disease activity at 24 weeks after the initiation of tofacitinib treatment with or without a concomitant use of MTX. **b** The proportion of disease activity at 24 weeks after the initiation of baricitinib treatment with or without a concomitant use of MTX. Disease activity was categorized as follows. DAS 28-ESR < 2.6 (remission), 2.6 to < 3.2 (LDA), 3.2–5.1 (MDA), > 5.1 (HDA). *MTX* methotrexate, *LOCF* last observation carried forward, *ESR* erythrocyte sedimentation rate, *DAS* disease activity score, *LDA* low disease activity, *MDA* moderate disease activity, *HDA* high disease activity

The proportions of disease activity at 24 weeks defined by the DAS28-ESR are shown in Fig. 2. There were no significant differences in the above indices between the patients with and without concomitant use of MTX in both groups.

### Drug retention and adverse events

**Fig. 3:**
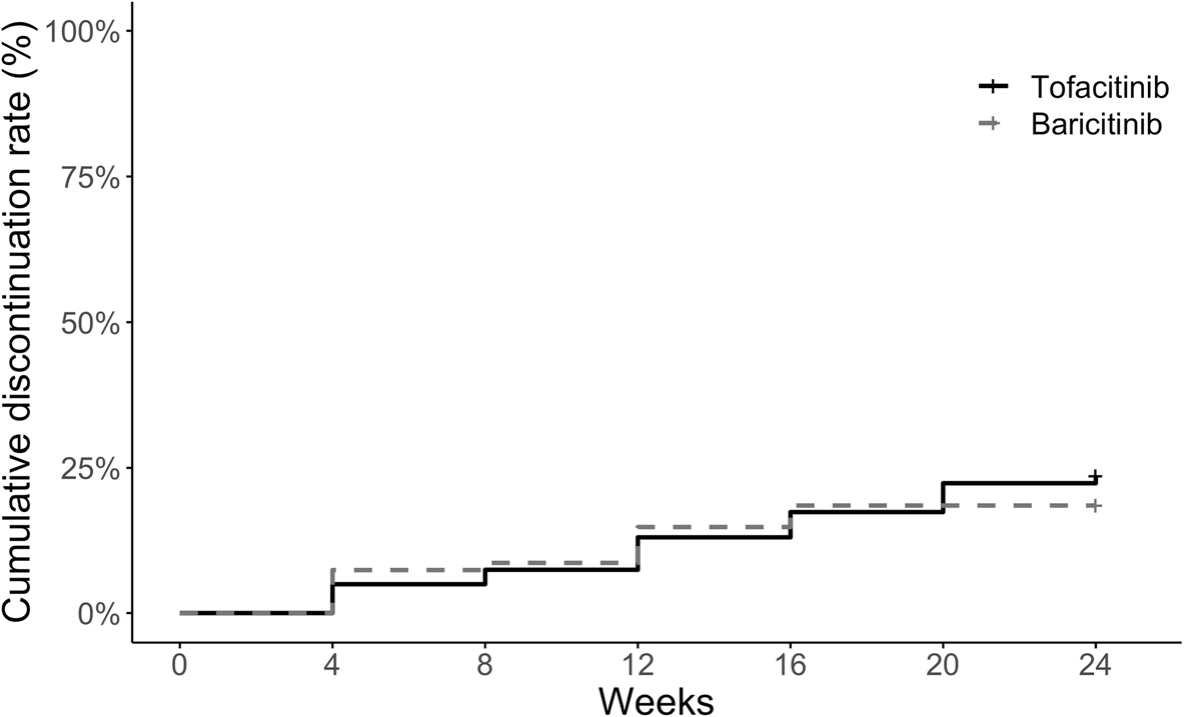
Estimated cumulative incidence of the discontinuation of tofacitinib and baricitinib treatment during the 24-week treatment period

There was no significant difference in the drug retention rate between the tofacitinib and baricitinib groups (adjusted hazard ratio for discontinuation,1.43; 95% CI, 0.76 to 2.67, tofacitinib vs baricitinib). The corresponding Kaplan-Meier plots of discontinuation are illustrated in Fig. 3. During the 24-week follow-up period, 38 patients (23.6%) were discontinued tofacitinib treatment and 15 patients (18.5%) were discontinued baricitinib treatment. The reasons for discontinuation were as follows. In the tofacitinib group, lack of efficacy (*n* = 17), request of the patient (*n* = 3), and an AE (pneumonia [*n* = 4], herpes zoster [*n* = 2], fungus infection, skin cancer, colon cancer, lung cancer, nausea [*n* = 4], interstitial pneumonia, vertigo, diarrhea, and hair loss) (*n* = 18) were the reasons. In the baricitinib group, lack of efficacy (*n* = 10) and an AE (pneumonia, herpes zoster, breast cancer, headache, and elevation of creatine kinase) (*n* = 5) were the reasons.

Table 3 summarizes the AEs experienced by the 32 patients in the tofacitinib group and the 14 patients in the baricitinib group. The incidence rate of AEs was not significantly different between the groups. The most common AE was infection (12.4% in the tofacitinib group, 13.6% in the baricitinib group). Among the infections, as expected, herpes zoster infection was the most frequent in both groups (5.6% in the tofacitinib group, 4.9% in the baricitinib group). Few neoplasms were seen in each group (3 in the tofacitinib group, 1 in the baricitinib group) during 24 weeks. The rate of herpes zoster was slightly higher in the tofacitinib group, and the difference was not significant. Moreover, the rates of other AEs such as infection and gastrointestinal disorder were also similar in each subgroup of the treatment groups.

**Table 3:** Adverse events

	Tofacitinib (*n* = 161)	Baricitinib (*n* = 81)	*P* value
All Events, *n* (%)	32 (19.9)	17 (21.0)	0.866
Infection	20 (12.4)	11 (13.6)	0.840
Herpes zoster	9 (5.6)	4 (4.9)	
Pneumonia	6 (3.7)	1 (1.2)	
Upper respiratory infection	4 (2.5)	3 (3.7)	
Callus infection		1 (1.2)	
Cytomegalovirus infection		1 (1.2)	
Fungus infection	1 (0.6)		
Sepsis		1 (1.2)	
Gastrointestinal disorder	6 (3.7)	2 (2.5)	0.720
Gastric ulcer		1 (1.2)	
Nausea	4 (2.5)	1 (1.2)	
Diarrhea	2 (1.2)		
Neoplasm	3 (1.9)	1 (1.2)	> 0.999
Breast cancer		1 (1.2)	
Colon cancer	1 (0.6)		
Skin cancer	1 (0.6)		
Lung cancer	1 (0.6)		
Others	3 (1.9)	3 (3.7)	0.405
Hair loss	1 (0.6)		
Vertigo	1 (0.6)	1 (1.2)	
Headache		1 (1.2)	
Interstitial pneumonia	1 (0.6)		
Elevation of creatine kinase		1 (1.2)	

### Comparison of factors contributing to a good clinical response between tofacitinib and baricitinib treatment

We next investigated the factors that contribute to the clinical responses to baricitinib and tofacitinib. The baseline characteristics that predict the achievement of DAS28-ESR-low disease activity (LDA) in the univariate analysis were as follows (Table 4): in the tofacitinib group, the concomitant use of oral steroid, the DAS28-ESR at the time of treatment initiation, and the value of mHAQ; in the baricitinib group, the concomitant use of an oral steroid, the number of b/tsDMARDs previously used, the DAS28-ESR at the time of treatment initiation, and the value of mHAQ. Among these factors, the results of the multivariable logistic analysis demonstrated that the concomitant use of an oral steroid was independently associated with the achievement of DAS-LDA in the tofacitinib group, whereas, in the baricitinib group, the multivariable analysis identified the significant association between the association of DAS28-ESR-LDA achievement and the number of b/tsDMARDs previously used, and the DAS-ESR at the time of treatment initiation.

**Table 4:** Independent predictors for the achievement of LDA at 24 weeks in multivariable analysis

	Tofacitinib				Baricitinib			
Variables	Univariate model		Multivariable model		Univariate model		Multivariable model	
	OR (95% CI)	*P* value	OR (95% CI)	*P* value	OR (95% CI)	*P* value	OR (95% CI)	*P* value
Age (per 1-year increase)	0.997 (0.969–1.026)	0.838	–		0.987 (0.951–1.023)	0.462	–	
Disease duration (per 1-year increase)	0.999 (0.965–1.034)	0.944	–		0.965 (0.919–1.014)	0.153	–	
Concomitant MTX use (yes/no)	1.02 (0.499–2.085)	0.957	–		1.869 (0.770–4.535)	0.165	–	
Concomitant oral steroid use (yes/no)	0.403 (0.203–0.799)	0.008*	0.470 (0.232–0.953)	0.035*	0.412 (0.167–1.013)	0.050	0.339 (0.102–1.129)	0.073
Number of previous use of b/tsDMARDs (per drug)	0.882 (0.714–1.09)	0.240	–		0.687 (0.522–0.905)	0.005*	0.700 (0.504–0.971)	0.026*
Inadequate response of another JAK inhibitor (yes/no)					0.511 (0.194–1.341)	0.167	–	
DAS28-ESR at baseline (per 1 increase)	0.798 (0.628–1.014)	0.061	0.882 (0.678–1.147)	0.345	0.395 (0.247–0.633)	< 0.001*	0.395 (0.225–0.694)	< 0.001*
mHAQ (per 1 increase)	0.603 (0.366–0.996)	0.039*	0.745 (0.424–1.304)	0.294	0.322 (0.155–0.667)	< 0.001*	0.697 (0.301–1.611)	0.396
ACPA positive (yes/no)	1.286 (0.568–2.912)	0.543	–		0.426 (0.138–1.314)	0.131	–	
RF positive (yes/no)	1.687 (0.733–3.886)	0.207	–		0.429 (0.115–1.599)	0.198	–	

*OR* odds ratio, *95% CI* 95% confidence interval, *MTX* methotrexate, *b/tsDMARDs* biological and/or targeted synthetic disease-modifying antirheumatic drugs, *JAK* Janus kinase, *DAS* disease activity score, *mHAQ* modified Health Assessment Questionnaire, *ESR* erythrocyte sedimentation rate, *ACPA* anti-citrullinated protein antibodies, *RF* rheumatoid factor

*P < 0.05

## Discussion

If superiority or inferiority exists among tsDMARDs, it is very important to determine this information for the algorithm of RA treatment. Knowledge of the baseline variables that influence the treatment response to each tsDMARD is also useful information when selecting a tsDMARD toward the goal of achieving better clinical outcomes, because in RA, many factors affect the treatment responses. For example, patients with shorter disease durations have shown better clinical outcomes with biological DMARDs compared to those with longer disease durations [20], and the serological status concerning autoimmune antibodies (RF and ACPA) is not only a prognostic factor but also a factor in treatment responses [21, 22]. Unfortunately, no head-to-head RCTs testing JAK inhibitors are available. In the present study, we compared the effectiveness and safety of two JAK inhibitors used to treat RA using multivariable analyses to avoid confounding. The findings obtained with our follow-up cohort in real medical practice demonstrated that tofacitinib and baricitinib had comparable efficacies and similar safety profiles but differences in the predictive factors that contribute to their treatment responses.

Our analyses revealed that 31.7% of the tofacitinib-treated group and 45.7% of the baricitinib-treated group achieved low disease activity as defined by the DAS28-ESR at 24 weeks, with no significant between-group difference. This result indicated that the two JAK inhibitors are effective and comparable in the daily clinical practice of treating RA patients who have various characteristics and treatment histories. Such a variety of clinical characteristics is different from the cohorts in most RCTs and thus has not been elucidated in RCTs.

It is crucial in RA treatment to know whether the efficacy of a drug depends on the concomitant use of MTX or not. In the present population, the tofacitinib and baricitinib treatments were effective even in patients without concomitant use of MTX. In those patients, the rates of LDA achievement were 30.8% in the tofacitinib group and 40.9% in the baricitinib group. These results are consistent with those of two phase III trials in which tofacitinib monotherapy and baricitinib monotherapy provided good clinical responses; the ACR 20/50/70 responses were 59.8/31.1/15.4 and 76.7/59.7/42.1, respectively [5, 13]. Moreover, based on those results, the 2019 EULAR recommendation for the management of RA stated that in patients who cannot use csDMARDs as comedication, IL-6 pathway inhibitors and tsDMARDs may have some advantages compared to other bDMARDs [3].

However, in our present investigation, although the difference was not significant, the patients with concomitant use of MTX showed better clinical efficacy compared to those without concomitant use of MTX in both the tofacitinib and baricitinib groups. The Δ (ΔDAS28-ESR from at baseline to at 24 weeks) values from the concomitant use of MTX to without the concomitant use of MTX were 0.35 in the tofacitinib group and 0.58 in the baricitinib group. And, our results also suggested that baricitinib treatment has more advantages in MTX-comedication therapy for the reduction of the DAS28-ESR score compared to tofacitinib treatment. The efficacy of concomitant therapy with MTX might be different for each JAK inhibitor. This point should be verified in studies with larger numbers of patients.

There was no significant difference in the retention rate between the present baricitinib and tofacitinib groups, as ~ about 80% of the patients continued treatment in both groups. These retention rates are comparable to those of the bDMARDs for which data were acquired from daily clinical practice [23–25]. In the present study, most of the instances of treatment discontinuation occurred before 12 weeks, and lack of efficacy was the most common discontinuation reason in both groups. This might reflect a treat-to-target strategy even in JAK inhibitor treatment, namely, “until the desired treatment target is reached, drug therapy should be adjusted at least every three months.” [26].

Adverse events occurred in 19.9% of the tofacitinib group and 21.0% of the baricitinib group in this study. As with other RCTs, herpes zoster infections were the most frequent AE (5.6% in the tofacitinib group and 4.9% in the baricitinib group). The incidence of herpes zoster infection was higher in the tofacitinib group, and this tendency has also been observed in RCTs. In an integrated analysis of RCTs and long-term extension studies, the incidence per 100 patient-years of herpes zoster was 4.0 in tofacitinib treatment and 3.3 in baricitinib treatment [27, 28]. Although the between-group difference in the herpes zoster infections in our study was not significant and the results of the integrated analysis cannot be compared directly because the patient backgrounds differ, we speculate that this difference might arise from the different selectivity for inhibition of the JAK pathway. Larger and longer-term studies are needed to examine this topic.

Interestingly, we observed that the predictors of treatment response differed by the type of JAK inhibitor. The concomitant use of an oral steroid was associated with the achievement of DAS-LDA in tofacitinib groups, whereas the number of b/tsDMARDs previously used was a significant factor only in the baricitinib group. The concomitant use of an oral steroid did not enhance the efficacy of the JAK inhibitors but decreased the efficacy of tofacitinib at 24 weeks. Considering this result and the potential harmful effects of oral steroids, the concomitant use of an oral steroid is not necessary for the treatment of RA using JAK inhibitors, and we should use the lowest possible dose of oral steroids for the shortest time as in the EULAR recommendation [3]. The result that the number of b/tsDMARDs previously used was associated with treatment response in the baricitinib group is consistent with another study in a real-world setting. Guideli et al. reported that the drug survival of baricitinib is higher in bDMARD-naïve patients [29]. Considering these results, the initiation of baricitinib at an earlier phase of RA might be better. To determine the optimal use of different JAK inhibitors in daily clinical practice, further analyses of predictors in real-world settings are necessary.

The treatment response to the JAK inhibitors among the patients who showed an inadequate response to another JAK inhibitor is also required information for the optimal use of JAK inhibitors. In the present baricitinib-treated group, 26 patients had been treated with tofacitinib. Although the multivariable analysis revealed no relationship between inadequate response to tofacitinib and the patients’ achievement of DAS28-ESR-LDA, the mean DAS28-ESR change from baseline to 24 weeks in the tofacitinib-treated patients was lower than that in the non-tofacitinib-treated patients (0.91, 1.27, respectively, *P* = 0.628). This result suggested the possibility of decreased efficacy of JAK inhibitors in the patients with inadequate response to another JAK inhibitor.

Limitations of this study are its small sample size and short observation period. In particular, the statistical reliability of the multivariable analysis that we performed in this study was not very strong because of the small samples. In addition, the study was carried out in a routine clinical practice situation, and we thus could not conduct a statistical power analysis about the sample numbers required for comparison before the study. We attempted to control for confounding using all available information and compared the efficacy and safety of JAK inhibitors. However, a perfect causal structure cannot be identified, and some confounders remain unmeasured; hence, residual confounding is not avoidable. In addition, we did not examine the effects on patient-reported outcomes (PROs) such as with the 36-item Short Form Health Survey (SF-36). The importance of PROs in RA management has been highlighted in order to reflect the patients’ satisfaction with their treatment and their role in making treatment decisions. Furthermore, the administration route of JAK inhibitors is oral, not a subcutaneous/intravenous injection. And, based on their regulation of multiple cytokines, JAK inhibitors have been thought to reduce pain in particular among the symptoms of RA [30–32]. The patients who are treated with JAK inhibitors might thus be satisfied with their treatments. We will analyze PROs in a future study.

## Conclusions

In conclusion, our findings demonstrated that tofacitinib and baricitinib had comparable continuing efficacies and safety profiles. The efficacy of the two drugs was almost the same even without the concomitant use of MTX. A lesser use of previous b/tsDMARDs contributed to the clinical response to baricitinib but not to tofacitinib. Comparisons performed with adjusting confounding can provide important and useful information about the optimal use of JAK inhibitors in the management of RA in real-world settings.

## Supplementary Information

**Additional file 1: Supplementary Table S1.** Comparison of baseline characteristics in patients with different backgrounds.

## Data Availability

The datasets used and/or analyzed during the current study are available from the corresponding author on reasonable request.

## Abbreviations

RA: Rheumatoid arthritis
bDMARDs: Biological disease-modifying antirheumatic dugs
JAK: Janus kinase
RCTs: Randomized controlled trials
PS: Propensity score
ACR: American College of Rheumatology
EULAR: European League against Rheumatism
RF: Rheumatoid factor
ACPA: Anti-citrullinated protein antibodies
mHAQ: Modified Health Assessment Questionnaire
JCR: Japan College of Rheumatology
DAS: Disease activity score
ESR: Erythrocyte sedimentation rate
SDAI: Simplified Disease Activity Index
CDAI: Clinical Disease Activity Index
AEs: Adverse events
MTX: Methotrexate
IQR: Interquartile range
b/ts: Biological or/and targeted synthetic
LOCF: Last observation carried forward
95% CI: 95% confidence interval
PROs: Patient-reported outcomes
SF-36: 36-item Short Form Health Survey
