# Supplementary material for: Real-world comparative effectiveness and safety of tofacitinib and baricitinib in patients with rheumatoid arthritis
Source: Arthritis Res Ther. 2021 Jul 23;23:197. doi: 10.1186/s13075-021-02582-z (PMC8299678; doi:10.1186/s13075-021-02582-z)
Supplement: Supplementary file 1 — Additional file 1: Supplementary Table S1. Comparison of baseline characteristics in patients with different backgrounds. [file 13075_2021_2582_MOESM1_ESM.docx]

**Supplementary Table S1 Comparison of baseline characteristics in patients with different backgrounds**

|  | Tofacitinib | | | Baricitinib | | |
| --- | --- | --- | --- | --- | --- | --- |
|  | Concomitant use of MTX | No concomitant use of MTX | P value | Concomitant use of MTX | No concomitant use of MTX | P value |
| Number of patients | 109 | 52 |  | 37 | 44 |  |
| Female, n (%) | 88 (80.7) | 45 (86.5) | 0.505 | 32 (86.5) | 36 (81.8) | 0.763 |
| Age (years) | 65 [54-71] | 69 [63-77] | *0.005 | 58 [49-70] | 71 [65-77] | *<0.01 |
| Duration of RA (year) | 11 [5-18] | 15 [8-20] | *0.042 | 9 [4-16] | 12 [3-20] | 0.446 |
| Concomitant oral steroid use, n (%) | 59 (54.1) | 27 (51.9) | 0.866 | 17 (46.0) | 21 (47.7) | >0.999 |
| Mean oral steroid dose (mg/day) | 4.5 ±2.63 | 5.6 ± 2.80 | 0.063 | 4.94 ± 3.37 | 4.69 ± 2.90 | 0.947 |
| ACPA positive, n (%) | 82 (75.2) | 42 (80.8) | 0.549 | 28 (75.7) | 37 (84.1) | 0.407 |
| RF positive, n (%) | 83 (76.1) | 39 (72.0) | >0.999 | 31 (83.8) | 39 (88.6) | 0.538 |
| No prior use of b/tsDMARDs, n (%) | 31 (28.4) | 6 (11.5) | *0.017 | 6 (16.2) | 11 (25.0) | 0.416 |
| Number of previous use of b/tsDMARDs | 2 [03] | 2 [2-3] | *0.004 | 2 [1-4] | 2 [1-4] | 0.82 |
| DAS28-ESR | 5.26 [4.14-6.18] | 4.87 [3.86-6.04] | 0.519 | 4.82 [4.27-5.74] | 5.22 [4.00-6.23] | 0.680 |
| SDAI | 22 [14-32] | 19 [14-28] | 0.535 | 17 [12-28] | 21 [15-30] | 0.409 |
| CDAI | 19 [12-30] | 19 [13-28] | 0.932 | 15 [11-26] | 18 [14-29] | 0.289 |
| mHAQ | 0.50 [0-1.0] | 1.0 [0.3-1.6] | *0.01 | 0.4 [0.1-1.0] | 1.0 [0.3-1.6] | *0.025 |

Data are median [interquartile range] unless otherwise indicated.

*RA* rheumatoid arthritis, *MTX* methotrexate, *ACPA* anti-citrullinated protein antibodies, *RF* rheumatoid factor*, b/tsDMARDs* biological and/or targeted synthetic disease-modifying antirheumatic drugs, *DAS* disease activity score, *ESR* erythrocyte sedimentation rate, *SDAI* simplified disease activity index, *CDAI* clinical disease activity index, *mHAQ* modified health assessment questionnaire *P<0.05 versus concomitant use of MTX in baricitinib group.
